# Molecular subtyping for source tracking of *Escherichia coli* using core genome multilocus sequence typing at a food manufacturing plant

**DOI:** 10.1371/journal.pone.0261352

**Published:** 2021-12-23

**Authors:** Ayaka Nakamura, Hajime Takahashi, Maki Arai, Tomoki Tsuchiya, Shohei Wada, Yuki Fujimoto, Yoshiomi Shimabara, Takashi Kuda, Bon Kimura

**Affiliations:** 1 Department of Food Science and Technology, Tokyo University of Marine Science and Technology, Minato-ku, Tokyo, Japan; 2 Nichirei Foods Incorporation, Chuo-ku, Tokyo, Japan; 3 Nichirei Corporation, Chuo-ku, Tokyo, Japan; University of Texas at San Antonio, UNITED STATES

## Abstract

When harmful bacteria are detected in the final product at a food manufacturing plant, it is necessary to identify and eliminate the source of contamination so that it does not occur again. In the current study, the source of contamination was tracked using core genome multilocus sequence typing (cgMLST) analysis in cases where *Escherichia coli* was detected in the final product at a food manufacturing plant. cgMLST analysis was performed on 40 strains of *E*. *coli* collected from the environment [floor (26 strains), drainage ditch (5 strains), container (4 strains), post-heating production line (1 strain)] and products [final product (3 strains) and intermediate product (1 strain)]. In total, 40 *E*. *coli* isolates were classified into 17 genogroups by cgMLST analysis. The 4 *E*. *coli* strains isolated from the intermediate and final products were classified into two genogroups (I and II). Certain isolates collected from the environment also belonged to those genogroups, it was possible to estimate the transmission of *E*. *coli* in the manufacturing plant. Thus, the dynamics of *E*. *coli* in the food manufacturing location were clarified by using cgMLST analysis. In conclusion, our results indicate that cgMLST analysis can be effectively used for hygiene management at food manufacturing locations.

## Introduction

*Escherichia coli* is mainly found in the intestinal tracts of animals [1, 2]; moreover, as *E*. *coli* presumably adheres to food as a result of fecal contamination, such contamination is widely used as a hygiene index for water and food products [3–5]. In Japan, the standard criteria for various processed products include the absence of *E*. *coli* and coliform bacteria in the final products [6]. Products that do not meet the standard criteria cannot be shipped to the market; this adversely affects food companies, causing economic loss and hampering the reputation of the companies. To avoid this situation, most food companies routinely conduct microbiological tests, such as wiping inspections of the production lines and sampling inspections. If *E*. *coli* is detected in the final product, the source of the contamination must be identified, and measures must be taken to eliminate the source of contamination through intervention strategies such as sanitization practices.

The strain typing method is commonly used to identify the source and route of microbial contamination [7–12]. DNA sequences of bacteria exhibit remarkable variation and diversity at the strain level even within the same species. Therefore, it is possible to clarify the source of contamination by distinguishing between bacteria isolated from food manufacturing locations at the strain level [7, 13]. The pulsed-field gel electrophoresis (PFGE) method has been widely used in epidemiological studies for strain typing [14–17]; however, it is difficult for food companies to use this method because of its complicated operation [16, 18]. For this reason, simple PCR-based methods, such as the randomly amplified polymorphic DNA (RAPD) method, and automated typing methods, such as ribotyping, have been widely used by food companies [10, 15, 19, 20]. Nevertheless, the reproducibility of the RAPD method is low [21], and it is often difficult to compare data between different production facilities or with previously obtained data. Furthermore, the high cost of the equipment and pre-packaged disposable kits for ribotyping is problematic [19, 21].

In recent years, the multilocus sequence typing (MLST) method, which focuses on the differences in DNA sequences, has been used for subtyping [22]. This method assigns different allele numbers to the differences in sequences of 5–7 housekeeping genes and distinguishes between the strains using the allele patterns obtained [23]. The MLST method presents a remarkable advantage in reproducibility because it recognizes the differences in sequences in multiple regions. Moreover, the sequence type (ST) obtained from the allele pattern is a simple number that can be compared between facilities and with previous isolates; however, it is necessary to individually obtain sequences for 5–7 gene regions, which is laborious and costly when performed via the Sanger method. Thus, it is not recommended for situations in which numerous samples need to be analyzed, particularly in the food industry.

The recent development of next-generation sequencing and its widespread use have made it possible to obtain abundant genomic information regarding bacterial strains at a relatively low cost and in a short time [13, 24]. As a result, core genes—common genes among different strains in the same species—were defined by various researchers, and the core genome MLST (cgMLST) method was developed as a subtyping method using these core genes [25–28]. Compared to the conventional MLST method, which targets approximately 5–7 housekeeping genes, the cgMLST method has a high strain discrimination capability because the number of target loci could reach several thousand [25]. Presently, this method is used mainly in epidemiological studies [29–31]. However, to the best of our knowledge, there are few reports using cgMLST analysis as a subtyping method for source tracking in food manufacturing plants [32, 33].

The purpose of this study was to evaluate the effectiveness of using cgMLST analysis for hygiene management at food manufacturing plants. For cases where *E*. *coli* was detected in the final product at the food manufacturing location, cgMLST analysis was performed for the *E*. *coli* isolates to identify the source of contamination. For comparison, classical MLST analysis and ribotyping analysis were also performed.

## Materials and methods

### Sample collection

A detection test for *E*. *coli* was performed on a total of 987 samples (wiping test: 822 samples, sampling test: 165 samples). For wiping test, 100 cm^2^ squares were wiped at multiple locations (floor, drainage ditch, container, equipment, and production line) in the food factory using a 3M ™ Quick Swab (3M Japan Limited, Tokyo, Japan). The wiping swab was suspended in the Letheen broth included in the kit and was used as the sample solution. Simultaneously, sampling inspections of intermediate and final products were conducted as follows. Phosphate-buffered saline (90 mL) was added to 10 g of the product, and the sample solution was homogenized at 480 rpm for 30 s using a homogenizer (AS ONE Corporation, Osaka, Japan). Thereafter, 1 mL of the suspension was inoculated onto a 3M Petrifilm ™ *E*. *coli* measurement plate (SEC plate) (3M Japan) and cultured at 42.0°C ± 1.0°C for 24 ± 2 h. After culturing, typical blue-green colonies were picked and streaked on eosin methylene blue (EMB) agar (Merck, Darmstadt, Germany) and cultured at 35°C ± 1.0°C for 24 ± 2 h. Colonies with a black metallic luster were identified as *E*. *coli*. Of the strains identified as *E*. *coli*, 40 strains that were estimated to be important for understanding the dynamics of the *E*. *coli* at this plant based on information such as the date and location of isolation, and were therefore selected for subsequent analysis.

### DNA extraction

A total of 40 *E*. *coli* strains were grown in trypticase soy broth (TSB; Becton, Dickinson and Company, Franklin Lakes, NJ) overnight at 37°C. DNA was extracted using the phenol–chloroform and ethanol precipitation [34]. Briefly, a 1-ml sample of enriched culture was centrifuged at 15,000 × g for 3 min, the bacterial cells were incubated in 567 μL of Tris–EDTA buffer containing lysozyme (5 mg /ml) for 1 h at 37°C, and cells were lysed by adding 30 μL of 10% (w/v) sodium dodecyl sulfate and 3 μl of proteinase K (20 mg/μL) followed by incubation for 1 h at 37°C. Next, 100 μl of 5 M NaCl was added, and DNA was extracted with chloroform–isoamyl alcohol (24:1) followed by phenol–chloroform–isoamyl alcohol (25:24:1). DNA was then precipitated with isopropanol, washed with 70% ethanol, and dried. Purified DNA samples were resuspended in Tris-EDTA buffer and used as DNA templates.

### Whole genome sequencing

In total, 40 *E*. *coli* strains were subjected to whole genome sequencing (WGS). WGS was outsourced to Bioengineering Lab. Co., Ltd. (Kanagawa, Japan). First, the DNA was fragmented using an ultrasonicator (Covaris Inc, MA, USA) to a fragment length of 400 bp. The library preparation was conducted according to the manual using a MGIEasy Universal DNA Library PrepSet (MGI Tech Co., Ltd, Shenzhen, China). The concentration of the prepared library was measured using the Qubit 30 Fluorometer (Promega Co., WI, USA) and the Qubit dsDNA HS assay kit (Promega). The quality of the prepared library was confirmed using Fragment Analyzer and a dsDNA 915 Reagent Kit (Advanced Analytical Technologies, NY, USA). Circular DNA was prepared according to the manual using the created library and the MGIEasy Circularization Kit (MGI Tech Co.). A DNBSEQ G400RS high-throughput sequencing kit (MGI Tech) was used to prepare DNA nanoballs (DNBs) according to the manual. Sequencing analysis of the prepared DNBs was performed using DNBSEQ G400 under the condition of 2 × 150 bp.

### *De novo* assembly and quality controls

*De novo* assembly and quality control were performed using the Geneious software (Biomatters, Ltd., Auckland, New Zealand). First, the quality of the raw reads was checked using FastQC. All raw reads were preprocessed using BBMap version 38.43 tools [35], wherein the adapters were trimmed and reads with less than 50 bp were removed based on the read with a quality below Q30 using BBDuk.sh [35]. *De novo* assembly was carried out using SPAdes version 3.11.1, using default parameters [36]. The result of assembly is available in S1 Table.

### cgMLST analysis

cgMLST analysis was performed using the BioNumerics v7.6 (Applied Maths, Sint-Martens-Latem, Belgium) whole-genome sequencing application. Assembly-based allele calling was used to determine the STs, with the *E*. *coli*/*Shigella* Enterobase scheme [37]. A phylogenetic tree was constructed using the cgMLST profiles for 40 *E*. *coli* isolates. The trees were constructed using the unweighted pair group method with arithmetic mean (UPGMA) algorithm.

### *In silico* MLST analysis

*In silico* MLST was performed based on WGS data. MLST types were assigned based on the assembled reads according to the *E*. *coli*/*Shigella* Enterobase scheme. According to a seven-gene MLST scheme, *E*. *coli* isolates were classified into STs, sharing all seven alleles.

### Ribotyping

Automated ribotyping was performed using a RiboPrinter microbial characterization system (Dupont Qualicon, Wilmington, DE, USA), following the manufacturer’s instructions. Briefly, isolates were streaked onto trypticase soy agar plates (Becton Dickinson), and appropriate amounts of colonies were then used for the analysis. Following automated cell lysis, digestion of DNA with *EcoRI*, electrophoresis, transfer of fragments, hybridization with an *E*. *coli* rRNA operon probe, and detection of hybridized bands via chemiluminescence were carried out. The resulting ribotypes were grouped by pattern.

## Results and discussion

### Isolation site of *E*. *coli*

*E*. *coli* was detected in 40 samples obtained from the floor of the heating room, floor of the raw material processing room, floor of the chilling process room, floor of the washing room, drainage ditch, and containers (Table 1). The results indicated that *E*. *coli* contamination had spread throughout the factory. In general, *E*. *coli* attaches to raw materials such as meat and vegetables [4, 38], which in turn adhere to the production line equipment and instruments [39]. If *E*. *coli* contamination of the production line and equipment is not effectively eliminated during daily cleaning operations, it may remain on the lines and cause additional contamination. At the food manufacturing plant examined in this study, *E*. *coli* was detected in the final products despite the use of heat treatment, suggesting the possibility of cross-contamination of the product from the line.

**Table 1:** Details regarding the 40 strains of *E*. *coli* used for cgMLST analysis.

Site	Description	The number on the factory floor plan*	The number of strains
Floor	Raw material processing room	①	2
	Washing room	②	3
	Heating process room	③	15
	Chilling process room	④	6
Drain	Heating process room	③	5
Container	Storing intermediate products after heating	③	4
Line	Post-heating processing line	⑤	1
Product	Intermediate product	④	1
	Final product	⑥	2
	Final product	⑥	1

**Fig 1:**
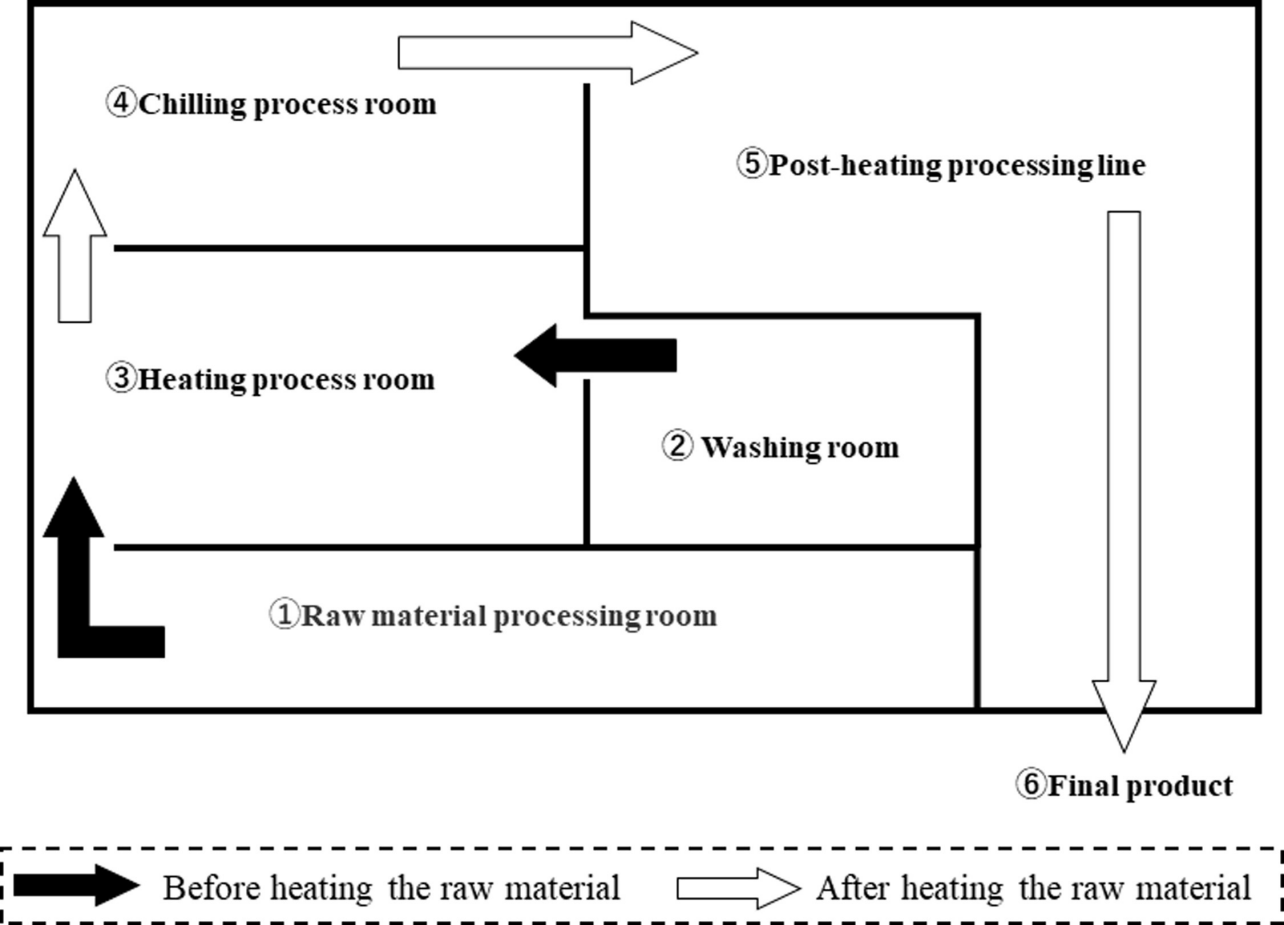
A simple floor plan of the production line for processed food (Chinese deli food) examined in this study.

* The number is the same as that displayed in Fig 1.

The food factory examined in this study has acquired FSSC 22000 certification and strives daily to manufacture safe food. This factory manufactures Chinese deli food, and this product is in the category of cooking with heat before eating. Therefore, even if *E*. *coli* slightly adheres to the product in this category, the risk of food poisoning is considered to be extremely low. However, it is important for food companies to reduce the frequency of *E*. *coli* contamination of final products to zero and aim for a higher level of hygiene management. Thus, in order to determine the source of the *E*. *coli* contamination, 40 *E*. *coli* isolates were subjected to cgMLST, classical MLST, and ribotyping analysis.

### Molecular typing analysis

**Fig 2:**
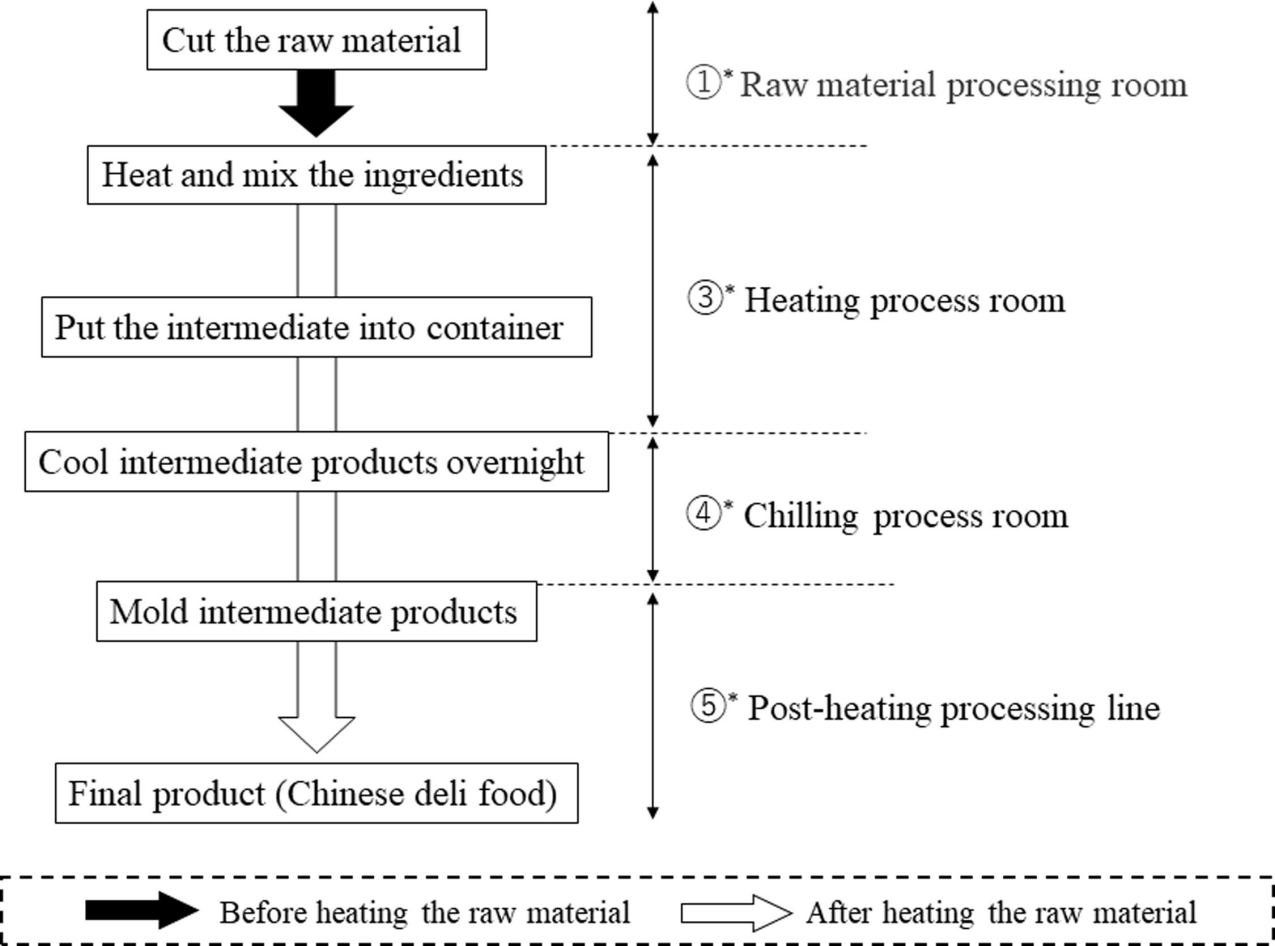
A schematic flow diagram of the food (Chinese deli food) processing line.

**Fig 3:**
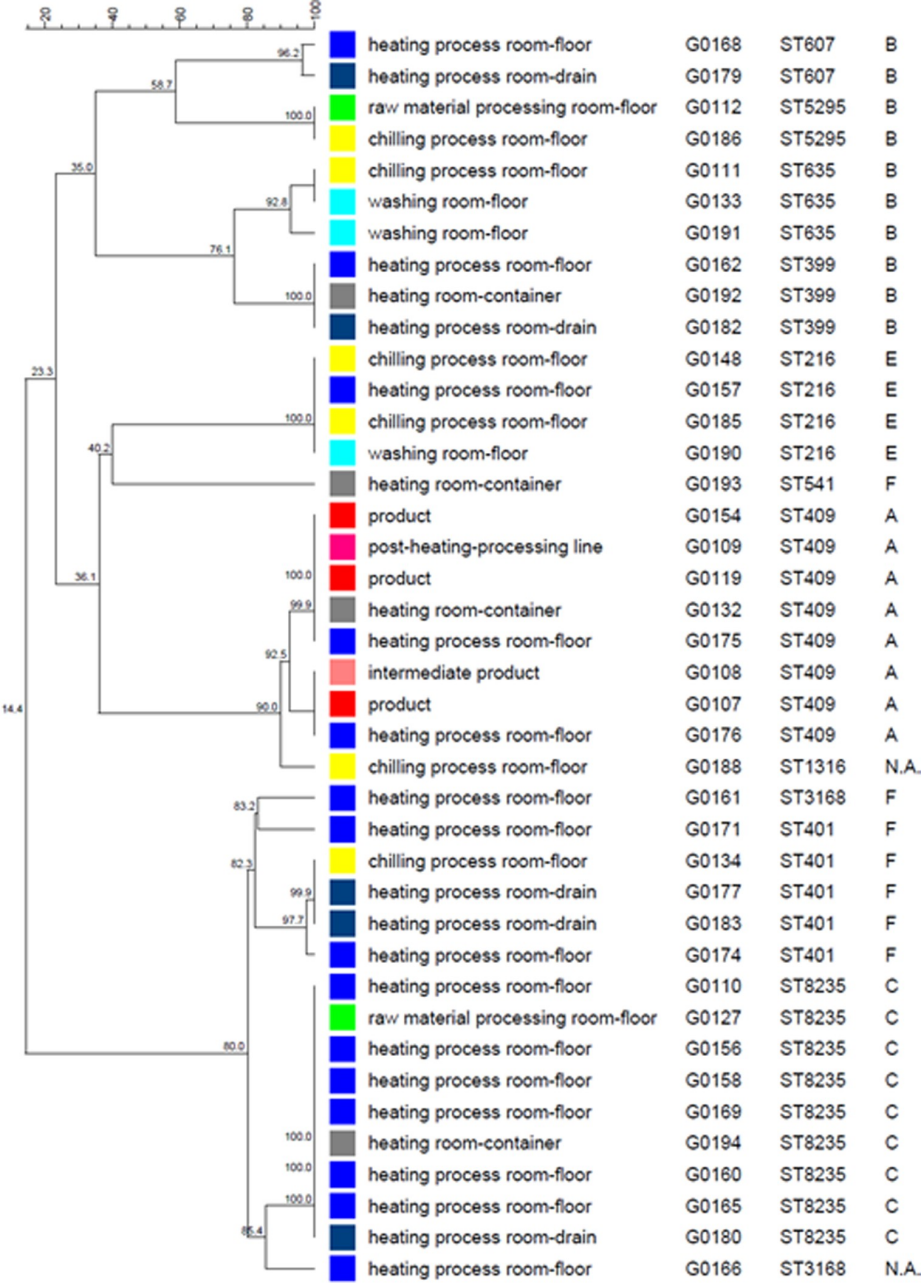
A phylogenetic tree constructed using the cgMLST profiles for 40 *E*. *coli* isolates from a food manufacturing plant. cgMLST profiles were generated using the 2513 core genes. The trees were constructed using the UPGMA algorithm. For comparison, the results of classical MLST and ribotyping patterns are also shown.

For a better understanding of the *E*. *coli* contamination source tracking, the floor plan of the factory is shown in Fig 1 and the manufacturing process for the product is shown in Fig 2. Based on the allele numbers obtained from the cgMLST analysis, phylogenetic trees were constructed using the cgMLST profiles for 40 *E*. *coli* isolates (Fig 3). The strains were classified into 17 patterns using the cgMLST analysis. For comparison, classical MLST analysis and ribotyping analysis were also performed, and the strains were classified into 11 and 7 patterns, respectively (Table 2). All strains that were classified into the same pattern by cgMLST analysis were also classified into the same pattern by ribotyping and classical MLST analysis. On the other hand, some strains classified into the same pattern by ribotyping analysis and classical MLST analysis were classified into different patterns by cgMLST analysis. From these results, the resolution of subtyping was the highest in cgMLST analysis, followed by classical MLST analysis and ribotyping analysis. In ribotyping analysis, bacterial DNA is fragmented with restriction enzymes and then subjected to electrophoresis; fragments containing genes encoding ribosomal RNA are detected among the separated fragments, and their patterns are examined [19]. Therefore, ribotyping focuses on the pattern of fragments containing ribosomal RNA genes in the truncated genomic sequence. On the other hand, cgMLST analysis is performed by comparing the nucleotide sequence of the core gene, that is, 2513 loci. The cgMLST analysis possessed the best discrimination ability because a wide range of regions on the genome were used for strain typing.

**Table 2:** Detailed information regarding the 40 *E*. *coli* strains used for subtyping and the results of classical MLST and ribotyping.

Strain number	Isolated location	Description	Isolated date	Sequence type (ST) of classical MLST	Pattern of riboprinting
G0107	Product	Product A	11-Jan-2020	409	A
G0109	Processing line	Post-heating	15-Feb-2020	409	A
G0154	Product	Product B	19-Feb-2020	409	A
G0108	Intermediate product	Product B	27-Feb-2020	409	A
G0111	Floor	Chilling process room	4-Mar-2020	635	B
G0110	Floor	Heating process room	4-Mar-2020	8235	C
G0112	Floor	Raw material processing room	9-Apr-2020	5295	B
G0119	Product	Product A	17-Apr-2020	409	A
G0132	Container	Heating process room	20-Apr-2020	409	A
G0133	Floor	Washing room	20-Apr-2020	635	B
G0127	Floor	Raw material processing room	20-Apr-2020	8235	C
G0148	Floor	Chilling process room	20-Apr-2020	216	E
G0134	Floor	Chilling process room	20-Apr-2020	401	F
G0156	Floor	Heating process room	21-May-2020	8235	C
G0157	Floor	Heating process room	21-May-2020	216	E
G0158	Floor	Heating process room	29-May-2020	8235	C
G0160	Floor	Heating process room	29-May-2020	8235	C
G0161	Floor	Heating process room	29-May-2020	3168	F
G0175	Floor	Heating process room	5-Jun-2020	409	A
G0176	Floor	Heating process room	5-Jun-2020	409	A
G0162	Floor	Heating process room	5-Jun-2020	399	B
G0182	Drainage ditch	Heating process room	5-Jun-2020	399	B
G0192	Container	Heating process room	5-Jun-2020	399	B
G0168	Floor	Heating process room	5-Jun-2020	607	B
G0179	Drainage ditch	Heating process room	5-Jun-2020	607	B
G0191	Floor	Chilling process room	5-Jun-2020	635	B
G0186	Floor	Chilling process room	5-Jun-2020	5295	B
G0165	Floor	Heating process room	5-Jun-2020	8235	C
G0169	Floor	Heating process room	5-Jun-2020	8235	C
G0180	Drainage ditch	Heating process room	5-Jun-2020	8235	C
G0194	Container	Heating process room	5-Jun-2020	8235	C
G0185	Floor	Chilling process room	5-Jun-2020	216	E
G0190	Floor	Washing room	5-Jun-2020	216	E
G0171	Floor	Heating process room	5-Jun-2020	401	F
G0174	Floor	Heating process room	5-Jun-2020	401	F
G0177	Drainage ditch	Heating process room	5-Jun-2020	401	F
G0183	Drainage ditch	Heating process room	5-Jun-2020	401	F
G0193	Container	Heating process room	5-Jun-2020	541	F
G0166	Floor	Heating process room	5-Jun-2020	3168	Not applicable to A to F
G0188	Floor	Chilling process room	5-Jun-2020	1316	Not applicable to A to F

Surprisingly, the strains isolated from the three final products and one intermediate product were classified into two genetically close genogroups by cgMLST analysis (Fig 3). The strains within each of these two genogroups are likely to be clones, as they exhibited sequence identity in all 2513 loci used for cgMLST analysis. These strains were classified into ST409 in classical MLST and pattern A in ribotyping (Table 2). This suggests that *E*. *coli* contamination of the final product may be caused by certain strains in the food manufacturing plant investigated in this study. Moreover, certain isolates collected from the environment also belonged to those genogroups (Fig 3). Here, G0154, G0109, G0119, G0132, and G0175 belonged to genogroup I, and G0108, G0107, and G0176 belonged to genogroup II. Genogroup I comprised *E*. *coli* isolates from the floor and container of the heating process room, container used in the heating process room, final product, and production line after the heating process. The container was used to store the intermediate products after the heating process in the chilling room (Tables 1 and 2 and Fig 2). Thus, contamination of this container with *E*. *coli* was extremely likely to cause *E*. *coli* contamination of the intermediate product. Containers are usually stacked from the floor in the heating process room. Moreover, *E*. *coli* with the same genotype as that found on the container was also isolated from the floor of the heating process room. From these results, it is presumed that the *E*. *coli* was transmitted from the floor of the heating process room to the final products via the container. In addition, *E*. *coli* of the same genotype was isolated from the production line after the heating process, suggesting the possibility of cross-contamination to other products. Similarly, for genogroup II, the genotypes of the isolates from the floor of the heating process room and the *E*. *coli* isolates from intermediate and final products were identical, indicating that the *E*. *coli* strains from the heating process room may have cross-contaminated the products after heating.

Furthermore, focusing on the isolation dates of each *E*. *coli* isolate, *E*. *coli* strains of the same genotype, G0176 and G0107, were isolated about 5 months apart. This suggests that *E*. *coli* strains that are resistant to environmental stress may have remained in the facility for a long period and repeatedly contaminated the products. In both genogroups I and II, the source of *E*. *coli* contamination was the floor of the heating process room, and thorough cleaning of this area, in particular, is necessary.

Classical MLST and ribotyping also clustered the contaminated products with the same sources as cgMLST in this study. This indicates that it is possible to obtain the same results as the cgMLST analysis, even when using classical MLST and ribotyping. However, looking at other strains within clusters, some were divided into different groups by cgMLST analysis but were classified into the same groups by classical MLST and ribotyping. In such cases, it may be difficult to estimate the dynamics of *E*. *coli* from the results of ribotyping and classical MLST analysis alone. If only classical MLST and ribotyping analyses are used, it is not possible to know if the patterning is the best resolution to estimate the source of contamination, which means that there is always a risk of misinterpreting the results.

It was observed that *E*. *coli* isolates from the same wiping location were not necessarily classified into the same genotype (Fig 3) and that several *E*. *coli* strains with different genotypes, that is, strains of different origin, were present in the same location. The greatest diversity in genotypes of the isolates was observed on the floor of the chilling room, where intermediate products are refrigerated, and the six *E*. *coli* isolates were classified into five genotype patterns. Those isolates had the same genotype as the *E*. *coli* strains isolated from the upstream side of the manufacturing process, such as the floors of the raw material processing room, heating process room, and washing room, and the drain of the heating process room. This suggested that the *E*. *coli* contamination was transmitted from upstream to downstream in the manufacturing process.

In this study, we did not investigate in the draft genomic data which genes are involved in the persistence of *E*. *coli* in the food processing plant and make a difference in the actual phenotype. Identifying genetic markers that are related to the persistence of *E*. *coli* in the factory leads to knowledge of the properties of *E*. *coli* and is especially useful for eliminating them from the factory. Therefore, in the future, it will be necessary to identify genes corresponding to various stresses to predict the properties of *E*. *coli*.

## Conclusion

This study evaluated the usefulness of cgMLST analysis, which can discriminate between strains at the clonal level, as a hygiene control method in food manufacturing zones. Ribotyping and classical MLST analysis were also performed for comparison. As a result, cgMLST analysis has the highest strain discrimination ability, and it was possible to estimate the source of contamination of products by undesirable bacteria. To our knowledge, only a few studies have reported the use of cgMLST analysis for estimating the source of contamination of undesirable bacteria in food production locations [32, 33]. In the future, the adoption of cgMLST analysis as one of the various sanitization management methods implemented at food manufacturing zones is expected to contribute to further improvement in sanitization controls.

## Supporting information

S1 Table The result of assembly using SPAdes. (DOCX)
