# Supplementary material for: Molecular subtyping for source tracking of Escherichia coli using core genome multilocus sequence typing at a food manufacturing plant
Source: PLoS One. 2021 Dec 23;16(12):e0261352. doi: 10.1371/journal.pone.0261352 (PMC8699670; doi:10.1371/journal.pone.0261352)
Supplement: S1 Table — (DOCX) [file pone.0261352.s001.docx]

**S1 Table. The result of Assembly using SPAdes.**

| Strain no. | Total reads | number of | min length(bp) | median length(bp) | Mean length(bp) | Max length(bp) | N50 length | number of contigs>=N50 | length sum(bp) |
| --- | --- | --- | --- | --- | --- | --- | --- | --- | --- |
| G0185 | 3121512 | 297 | 128 | 1583 | 16735 | 170342 | 76631 | 23 | 4970554 |
| G0148 | 1110983 | 277 | 128 | 2453 | 17900 | 175595 | 62885 | 23 | 4958546 |
| G0190 | 3036275 | 280 | 128 | 1587 | 17638 | 187331 | 80749 | 22 | 4938854 |
| G0157 | 2781560 | 261 | 128 | 1926 | 18877 | 170306 | 80920 | 22 | 4927086 |
| G0162 | 3011679 | 217 | 128 | 1238 | 23603 | 270562 | 101290 | 16 | 5121885 |
| G0182 | 3890551 | 232 | 128 | 1166 | 21913 | 269951 | 101293 | 16 | 5083890 |
| G0192 | 2527039 | 255 | 128 | 1154 | 20449 | 270434 | 87322 | 18 | 5214744 |
| G0134 | 1138005 | 424 | 128 | 756 | 12155 | 174968 | 66258 | 27 | 5154140 |
| G0174 | 2254030 | 358 | 128 | 673 | 14257 | 267422 | 80472 | 20 | 5118485 |
| G0171 | 2411669 | 336 | 128 | 783 | 15586 | 293334 | 82333 | 17 | 5237111 |
| G0177 | 2869924 | 514 | 128 | 441 | 10083 | 174990 | 69344 | 25 | 5182995 |
| G0183 | 3249441 | 384 | 129 | 823 | 13148 | 175102 | 69798 | 24 | 5049179 |
| G0109 | 1106227 | 203 | 128 | 2821 | 23931 | 277581 | 100290 | 17 | 4858093 |
| G0108 | 1036871 | 213 | 133 | 2591 | 22721 | 260286 | 94541 | 16 | 4839649 |
| G0175 | 2856013 | 347 | 127 | 741 | 14999 | 264570 | 85376 | 18 | 5204948 |
| G0176 | 2496967 | 251 | 128 | 844 | 19174 | 259853 | 94170 | 16 | 4812733 |
| G0132 | 1303570 | 284 | 128 | 1603 | 18379 | 264566 | 82977 | 20 | 5219699 |
| G0107 | 1105162 | 201 | 128 | 3350 | 23909 | 261701 | 93951 | 16 | 4805907 |
| G0119 | 1018506 | 201 | 128 | 4314 | 24177 | 264567 | 86710 | 18 | 4859687 |
| G0154 | 1672425 | 228 | 128 | 1529 | 21348 | 264568 | 105532 | 16 | 4867535 |
| G0193 | 1709420 | 437 | 128 | 948 | 12123 | 196271 | 58268 | 29 | 5297923 |
| G0168 | 2621411 | 292 | 128 | 518 | 17964 | 546251 | 180233 | 9 | 5245706 |
| G0179 | 2773080 | 421 | 128 | 362 | 12728 | 368148 | 178144 | 10 | 5358684 |
| G0111 | 1177382 | 280 | 128 | 1601 | 17871 | 209682 | 74843 | 22 | 5004036 |
| G0133 | 1018994 | 216 | 128 | 2172 | 22777 | 209657 | 83210 | 18 | 4919860 |
| G0191 | 2634355 | 318 | 128 | 1054 | 16466 | 322702 | 84173 | 19 | 5236471 |
| G0188 | 3226897 | 405 | 128 | 677 | 12831 | 282502 | 82739 | 21 | 5221170 |
| G0161 | 10437086 | 320 | 128 | 725 | 15694 | 255159 | 96636 | 16 | 5022078 |
| G0166 | 2891474 | 511 | 128 | 680 | 10450 | 241846 | 75889 | 22 | 5339955 |
| G0186 | 3238194 | 454 | 128 | 474 | 12283 | 243350 | 82992 | 23 | 5576820 |
| G0112 | 1194811 | 251 | 128 | 1476 | 20256 | 242951 | 99746 | 18 | 5084368 |
| G0127 | 1065826 | 399 | 128 | 1371 | 12935 | 247909 | 68802 | 22 | 5161315 |
| G0160 | 3532542 | 357 | 128 | 1336 | 14116 | 249791 | 70817 | 22 | 5039480 |
| G0169 | 2291391 | 397 | 128 | 1127 | 12851 | 248161 | 70160 | 22 | 5101890 |
| G0165 | 2869667 | 402 | 128 | 1046 | 12719 | 248161 | 73274 | 21 | 5113152 |
| G0156 | 2739678 | 413 | 128 | 1196 | 12573 | 247906 | 70168 | 22 | 5192908 |
| G0158 | 2745834 | 356 | 128 | 1404 | 13896 | 245244 | 73501 | 20 | 4947000 |
| G0110 | 999430 | 407 | 128 | 1082 | 12598 | 248464 | 68804 | 22 | 5127540 |
| G0180 | 3010065 | 815 | 128 | 667 | 7235 | 245290 | 53679 | 31 | 5896623 |
| G0194 | 1761985 | 366 | 128 | 1164 | 13567 | 249791 | 69077 | 21 | 4965887 |
